# Exercise as a community treatment for obesity and the metabolic disease epidemic

**DOI:** 10.1017/cts.2023.631

**Published:** 2023-09-15

**Authors:** John Scholler, Jeffrey S. Otis

**Affiliations:** Department of Kinesiology and Health, Georgia State University, Atlanta, GA, USA

**Keywords:** Metabolic syndrome, obesity, resistance training, aerobic training, combined training, community

## Abstract

The obesity epidemic has continued to rise at an alarming rate and has increased health complications in children, adolescents, and adults. Resistance training, aerobic training, and a combination of the two have been shown to be effective at reducing excess adiposity and improving outcomes of obesity. The continued development of programs, community centers, or medical exercise facilities prescribing these treatments is nearing an absolute necessity to slow the advancing nature of obesity and related metabolic diseases. In this brief review, we summarize the effectiveness of these three training paradigms from a population-centric perspective.

## Introduction

The obesity epidemic continues to accelerate throughout the world and is a direct risk factor in developing metabolic diseases such as type II diabetes mellitus (T2DM) and metabolic syndrome (MS) [1]. Not only does this rise in obesity result in comorbid health conditions but is also shown to dramatically increase healthcare expenditure, estimating to cost $150 billion a year in the USA alone [2].

Resistance training (RT), aerobic training (AT), and a combination of the two (CT) have been shown to be effective at reducing excess adiposity and reversing obesity [3]. Utilizing exercise as a medical adjunct treatment alongside standard care can serve to reduce the overall cost of the conditions arising from obesogenic diseases or prevent their development outright [2]. The development of programs, community centers, or medical exercise facilities is vital to slow the advancing nature of obesity and related metabolic diseases.

However, there is a critical need to shift the paradigm of treatment from an individual-centered approach toward treating populations as a whole [4]. Accordingly, to better align future research on population-centric treatment protocols, a well-described directive must define, promote, and examine exercise interventions to best understand how to effectively develop treatment plans to impact larger populations. The hope is that this research can establish a foundation of criteria to shape future community programs and policies to attempt to end the vicious cycle of obesity and metabolic decay.

## Resistance Training (see Table 1)

RT utilizing free weights, machines, body weight exercise, and other equipment is designed to apply forces to an individual’s skeletal muscle system to drive muscular recruitment, strength, and hypertrophy. The process of acquiring these adaptations has been shown to improve biomarkers related to obesity, T2DM, and MS [5–10].

**Table 1.** Comparison of studies using resistance training (RT) to treat obesity, type II diabetes mellitus (T2DM), and metabolic syndrome (MS)

Author, date (Ref. #)	*n*	Study design, control (Y/N)	Population information	Intervention	Exercise prescription	Primary objective	Nutritional considerations	Observed outcomes
Davy et al., 2017 [5]	159	Randomized clinical trial,(Y) control group given RT but not SCT	Sedentary, elder overweight adults, diabetic, prediabetic, locally recruited with flyers, handouts, word of mouth, etc.	3 months, supervised RT followed by 3 months either SCT or standard care, 15 month follow-up performed	2x weekly on nonconsecutive days, total body RT performed, 1 set of each exercise with 8–12 repetitions maintaining form and effort until concentric failure	Influence of SCT on RT and possible enhancement of RT for adults with prediabetes	3-day diet recall at baseline and months 3, 9, and 15	−34% prevalence of prediabetes, +18% odds of NorGly with % increase of FFM, −BF%*, +LBM%*, −WC* (values not available as baseline to post treatment changes not reported)
Sigal et al., 2007 [8]	64	RCT with parallel arms, participants randomized to either RT, AT, CT, or control, (Y) CONT given stretching exercises	39–70 year old adults with T2DM, locally recruited with handouts, flyers, word of mouth, etc.	22 weeks of supervised RT	3x/week total body RT, progressing from 1 set to 3, performing maximum load to allow 7–9 repetitions	To compare and contrast impact of RT, AT, and CT have on HbA1c for T2DM	Maintenance caloric recommendations with dietary counseling from dietician at baseline, 3, and 6 months	HbA1c: −.38%, BM: −0.7 kg, BMI: −0.26, WC: −1.8 cm, LBM: +0.75 kg, BF%: −1.2%
Church et al., 2010 [9]	73	RCT with parallel arms (Y) CONT given stretching and relaxation exercises only	T2DM sedentary adults recruited from community via handouts, flyers, word of mouth, etc.	9 months of supervised RT	3x/week total body RT, 9 sets of 10–12 repetitions, load increased with successful set of 12 in back-to-back sessions	Examine the impact of similar duration RT, AT, and CT to see which modality has greatest impact on HbA1c and body composition	Weekly counseling with prescribed caloric intakes	HbA1c: −.19%, WC: −2.0 cm, BM: −0.2 kg, LBM: +0.9 kg, FM: −1.3
Minges et al., 2011 [10]	86	Retrospective cohort study, (N)	Adults with T2DM or at risk of developing T2DM that had previously enrolled in Lift for Life Program	24 weeks of supervised RT	3x/week total body RT performing 3 sets of 8–10 repetitions, load increased when 3 sets of 10 completed successfully	Evaluate effectiveness of Lift For Life RT program to improve T2DM biomarkers and anthropometrics	N/A	WC: −2.3 cm at wk 8, −2.5 cm at wk 16, and −4.9 cm at wk 24
Bateman et al., 2011 [11]	66	Randomized clinical trial, (N)	Sedentary, overweight, dyslipidemic adults with MS, recruited from community via flyers, handouts, word of mouth, etc.	8 months supervised RT	3x/week, 3 sets of 8–12 repetitions for 8 exercises, load increased 5 lbs after completing 12 reps in repeat sessions	Compare and contrast RT, AT, and CT on improving MS score and reducing comorbidities associated with MS	N/A	WC: +0.25 cm, FT: −0.37 mg/dl, BM: −.7 kg
Normandin et al., 2017 [12]	126	RCT, (Y) CONT group given same RT prescription but assigned maintenance calories	65–79-year-old, sedentary, overweight, or obese adults, recruited from community using flyers, handouts, word of mouth, etc.	5 month supervised RT	3x/week total body RT for 3 sets of 10 repetitions at 70% 1-RM, retest 1-RM every 4 weeks	To assess the difference in performing an RT program to improve metabolic health with/or without application of caloric restriction	63 randomized to prescribed CR diet rest were asked to follow eucaloric diet	BM: −0.1 and −4.9 kg*, CHO: +6.9 and −0.38 mg/dl, INS: −3.1* and −1.4 μIU/ml, HOMA-IR: −0.5 and −0.3 μIU/ml×mg/dl (listed as noCR and CR intervention, respectively)

AT = aerobic training; BF = body fat; BM = body mass; BMI = body mass index; CHO = carbohydrate; CONT = continuous; CR = caloric restriction; CT = combination of resistance training and aerobic training; FFM = fat free mass; FT = fasting triglycerides; HOMA-IR = homeostatic model assessment for insulin resistance; INS = insulin; LBM = lean body mass; MS = metabolic syndrome; N = no (not included in the study); RCT = randomized clinical trial; RM = repetition max; RT = resistance training; SCT = social cognitive theory; T2DM = type II diabetes mellitus; WC = waist circumference; Y = yes (included in the study).

Decreased waist circumference was the most commonly reported positive outcome of RT [6,9,10]. Glycemic control was improved by RT in several interventions [5,6] with null values only reported during unsupervised training [6]. Importantly, no studies reported a negative impact on glycemic control. RT had improved measures of body composition [5–7,9,10] and was only found to not be effective when dietary interventions were not also prescribed [8].

Interestingly, direct supervision of RT interventions appears directly related to effectiveness [5]. Another common moderator to exercise studies is the use of nutritional interventions aimed at modulating daily caloric intakes, either with maintenance or deficit caloric levels [6,10]. A single study [10] looked at RT comparatively with designed caloric restriction. The group that followed the reduced caloric intake saw significant improvements in waist circumference and body composition compared to controls. These two protocol designs provide key pieces to the efficacy of an RT program in terms of improving health conditions related to obesity and metabolic disease state and development.

The primary hurdles to program effectiveness are participant time commitments and funding availability. These two constraints on recruiting larger bases of a population cannot be ignored as improving markers for metabolic health in nearly every study correlated with researcher direct supervision of RT. Minges *et al*. [9], reported average cost per person was $13–$15 dollars. By extension, feasibility studies involving 5–10 times (∼500–1000 individuals) as many people would need to be designed to estimate how cost would be impacted by increasing sample sizes that greatly.

RT performed under direct supervision and dietary counseling has been shown to be beneficial in reducing obesity, reverting, or dampening severity of MS, and improving symptomology of T2DM. When RT is utilized without controlling for those moderating factors, the impact on clinical biomarkers and anthropometrics of importance is attenuated. While several factors may be critically important in the success of RT to treat those conditions, there were no negative impacts on those markers suggesting that RT will not negatively impact health status of populations of interest.

## Aerobic Training (see Table 2)

AT utilizes exercise to maintain a constant rate of muscle exertion to facilitate increases in a participant’s heart rate. Training approaches can be moderate (MIT-60% ∼ 80% of maximum) or a high-intensity approach (HIT-8%5 ∼ 100%). Several studies have indicated AT to be a successful treatment to improve body composition [6,7], reduce body weight [6–8,11,12], and improve glycemic control (e.g., reducing HbA1c [6–8] or fasting glucose levels) [11,12]. Phillips *et al*. [12] examined HIT on cardiometabolic markers of T2DM patients and reported success compared to MIT approaches. Therefore, the reductions seen in total training time using HIT approaches could be a more feasible treatment when applying AT to larger populations.

**Table 2.** Comparison of studies using aerobic training (AT) to treat obesity, type II diabetes mellitus (T2DM), and metabolic syndrome (MS)

Author, date, (Ref. #)	*n*	Study design/CONT (Y/N)	Population information	Intervention	Exercise prescription	Primary objective	Nutritional considerations	Observed outcomes
Sigal et al., 2007 [8]	60	RCT with parallel arms, participants randomized ot either RT, AT, CT, or control, (Y) CONT given stretching exercises	39–70-year- old adults with T2DM, locally recruited with handouts, flyers, word of mouth, etc.	22 weeks of supervised AT	3x/week treadmills or cycle ergometers, began with 15–20 mins @ 60% of HR_max_ and increased up to 45 mins @ 75%, polar HR monitors used	To compare and contrast impact of RT, AT, and CT have on HbA1c for T2DM	Maintenance caloric recommendations with dietary counseling from dietician at baseline, 3, and 6 months	HbA1c: −.51%*, BM: −2.2 kg*, WC: −.74 cm*, BMI: −.74*, FM: −1.84 kg, BF%: −1.0%, LBM: −.47 kg
Church et al., 2010 [9]	72	RCT with parallel arms, (Y) CONT given stretching and relaxation exercises only	T2DM sedentary adults recruited from community via handouts, flyers, word of mouth, etc.	9 months of supervised AT	Given 12 kcal/kg caloric expenditure per week, done within 50-80% VO_2max_, ACSM calorie expenditure used	T2DM sedentary adults recruited from community via handouts, flyers, word of mouth, etc.	Weekly counseling with prescribed caloric intakes	HbA1c: −19%, BM: −0.8 kg, WC: −1.6 cm, LBM: −0.4 kg, FM: −.7 kg
Bateman et al., 2011 [11]	73	Randomized clinical trial, (N)	Sedentary, overweight, dyslipidemic adults with MS, recruited from community via flyers, handouts, word of mouth, etc.	8 months supervised AT	Treadmills, ellipticals, or bike ergometers, 14 kcal/kg calorie expenditure per week, done between 65 and 80% VO_2peak_	Compare and contrast RT, AT, and CT on improving MS score and reducing comorbidities associated with MS	N/A	WC: −1.12 cm, FT: −.22 mg/dl, BM: −1.54 kg*
Chang et al., 2016 [13]	65	RCT, (Y) CONT given access to classes and equipment but not provided with support from researchers	Obese adults diagnosed with MS, recruited from community dwellings	6 months supervised AT with with hands-on staff help	3x/week of instructor guided 40-min aerobic exercise	Evaluate the impact of connection with providers has on success of exercise programs to treat MS	N/A	BM: −1.09 kg*, BMI: −.74*, WC: −3.63 cm*, FT: −5.84 mg/dl*
Phillips et al., 2017 [14]	210	RCT, (Y) non-exercise control group used as a comparator	Sedentary adults with impaired glucose tolerance and/or BMI > 27, recruited locally with flyers, handouts, word of mouth, etc.	6 months supervised AT using either 7x1 or 5x1 (rest work interval) HIIT	7x1 min cycling @100% of workload at VO_2max_ followed by 1 min rest, swapped for 5x1 minute program with later subjects	Assess short- duration HIIT training has for people with impaired glucose tolerance	N/A	BMI: −0.2 and −0.4, BM: −0.4 and +0 kg, FT −0.4 & −0.3 mmol/L-1, INS: −0.4 and −.8* pmol/l-1, HOMA-IR: −0.04 and .05* (values listed as 7x1 and 5x1 HIIT, respectively)

ACSM = American College of Sports Medicine; AT = aerobic training; BF = body fat; BM = body mass; BMI = body mass index; CONT = continuous; CT = combination of resistance training and aerobic training; FT = fasting triglycerides; FM = fat mass; HIIT = high intensity interval training; HOMA-IR = homeostatic model assessment for insulin resistance; HR_max_ = heart rate max; INS = insulin; LBM = lean body mass; MS = metabolic syndrome; N = no (not included in the study); RCT = randomized clinical trial; RT = resistance training; T2DM = type II diabetes mellitus; VO_2max_ = maximal oxygen consumption; VO_2peak_ = peak oxygen consumption; WC = waist circumference; Y = yes (included in the study).

Missing from many of these studies is incorporation of nutritional considerations when attempting to influence metabolic regulation for these populations [8,11,12]. The lack of nutritional control is somewhat perplexing when comparing the positive trends in blood flow reduction seen in these AT trials. AT may be more effective at improving those biomarkers when no change is presented in a participant’s dietary patterns which could be beneficial when working with larger populations. In parallel, the use of accelerometers and phone applications were successful at reducing the need for direct supervision, encouraged self-monitoring, and could prove helpful in larger sample sizes [11,12].

Overall, AT exercise prescriptions are effective at promoting positive changes in biomarkers of interest. While AT was able to confer similar improvements in body composition and glycemic control, it did not offer a similar enhancement in increasing lean body mass that was seen in RT. While improving muscular hypertrophy would aid to reduce the negative risk factors associated with obesity and related conditions, the lowered barrier of not requiring more skilled RT to improve an individual’s health poses a unique benefit of AT only prescriptions. An exercise program that is designed to optimally combat progressive obesity ideally would include improving body composition with the preservation of or increase in lean muscle tissue.

## Combined Training (see Table 3)

The combination of AT and RT is a successful intervention to improve glycemic control [6,7,13–15] and body composition [7,14,15]. CT outperformed both AT and RT exercise prescriptions alone in improving HbA1_c_ [7,8] and led to higher losses of body fat [10] at the end of the intervention when directly comparing them. HbA1_c_ was continually improved when CT was utilized as an intervention [13–15] and seen similarly with previous studies a congruent decrease in waist circumference [13–15]. CT was as equally effective at improving muscle hypertrophy as compared to RT alone and attenuated any decrements in fat free mass evident in AT alone.

**Table 3.** Comparison of studies using a combination of resistance training and aerobic training (CT) to treat obesity, type II diabetes mellitus (T2DM), and metabolic syndrome (MS)

Author, date (Ref. #)	*n*	Study design, CONT (Y/N)	Population information	Intervention	Exercise prescription	Primary objective	Nutritional considerations	Observed outcomes
Sigal et al., 2007 [8]	64	RCT with parallel arms, participants randomizedto either RT, AT, CT, or CONT, (Y) CONT given stretching exercises	39–70-year-old adults with T2DM	22 weeks of supervised CT	Same prescription of RT combined with AT (please see Tables 1 & 2 for exercise prescriptions)	To compare and contrast impact of RT, AT, and CT have on HbA1c for T2DM	Maintenance caloric recommendations with dietary counseling from dietician at baseline, 3, and 6 months	HbA1c: −.9%*, WC: −2.6 cm*, BMI: −.8*, LBM: −0.7 kg, BF%: −1%
Church et al., 2010 [9]	62	RCT with parallel arms, (Y) CONT given stretching and relaxation exercises only	T2DM sedentary adults	9 months of supervised CT	1x/week 10 kcal calorie expenditure between 50% and 80% VO_2max_, 2 RT sessions (see Table 1) performed for 1 set of 10–12 reps	Examine the impact of similar duration RT, AT, and CT to see which modality has greatest impact on HbA1c and body composition	Weekly counseling with prescribed caloric intakes	HbA1c: −.38%, BM: −1.5 kg, WC: −2.8 cm, LBM: +0 kg, FM: −1.7kg
Blüher et al., 2013 [16]	115	Cohort (N)	7–18-year-old children and adolescents with either impaired CHO metabolism, features of MS, or family history of obesity/T2DM that enrolled in school program	1 year structured and supervised AT and RT program along with intensive lifestyle intervention	AT and RT split up among 60–90 min guided exercise sessions, RT performed on Gymboy Teca®, AT on treadmills and stationary bikes	Test the validity or school program’s ability to decrease further incidence of T2DM/MS and reduce severity and also improve mental and physical well being of students	Classes offered to parents to improve quality of diets, direct counseling provided to parents and students	BM: +3.9 kg, BMI: −0.1, WC: +1.1 cm, BF%: −1%, HbA1c: −.25%, HOMA-IR: −0.1
Balducci et al., 2010 [17]	288	RCT, (Y) Provided with either physical activity counseling or standard treatment of T2DM	Sedentary T2DM patients enrolled in outpatient diabetes clinics	6 month supervised combined AT and RT program	150 mins/week guided mixed RT/AT sessions, 4 RT exercises performed and time matched AT, 3 stretching exercises prescribed as well	Examine efficacy of program’s CT program along with assistance of staff could decrease HbA1c of diabetics	Food diaries used to track food intake through intervention, prescribed 500 calorie deficit and macronutrient allowances per each subject	HbA1c: −30%*, FT: −.68 mg/dl, INS: −1.18 µU/mL*, HOMA-IR: −0.36, WC: −3.6 cm*, BMI: −.78*
Petrella et al., 2014 [18]	127	RCT, (Y) Active control used were given similar exercise prescription but not given access to mHealth app	18–70 yr old adults with ≥ 2 risk factors of MS	CT exercise prescription given with assessments at baseline, and weeks 12, 24, and 52. mHealth APP given to see enhancements of AT	AT performed 5-7x/week from 70% to 85% age estimated max HR to equal out to 30–60mins/day, RT done 2–4x/week designed to meet WHO RT guidelines in addition to meet subjects wants to facilitate adherence	To assess the impact of incorporating mHealth phone app alongside exercise prescriptions for MS	Nutritional counseling provided at each assessment along with 3-day food recall diaries	WC: −2.2 and −2.3 cm, FT: =0.04 and +0.07 mmol/L, HbA1c: −1.0 & −0.02 %, HOMA-IR: −0.18 and +0.23 (values listed for mHealth app group and active control, respectively)

APP = application; AT = aerobic training; BF = body fat; BMI = body mass index; CHO = carbohydrate; CT = combination of resistance training and aerobic training; FT = fasting triglycerides; FM = fat mass; MS = metabolic syndrome; CONT = continuous; HOMA-IR = homeostatic model assessment for insulin resistance; INS = insulin; LBM = lean body mass; N = no (not included in the study); RCT = randomized clinical trial; RT = resistance training; T2DM = type II diabetes mellitus; VO_2max_ = maximal oxygen consumption; WHO = World Health Organization; WC = waist circumference; Y = yes (included in the study).

Direct supervision of the RT component in CT ensures proper application of form and is correlated with clinical success. The inclusion of AT did not seem to diminish the positive impacts of RT and aided in improving cardiometabolic risk factors. Importantly, increasing lean body mass or decreasing body fat percentage and improving glucose tolerance was seen when using AT and RT both on the same day or on different days and thus provides flexibility during program prescription.

Participants achieve independence and potentially a reduced financial burden when provided supervised RT at a designated facility and completing AT outside of the facility. Further, this halved supervision approach (while maintaining effectiveness provides a clear advantage to CT for application to much larger study populations.

## Exercise as a Population Intervention

CT offers the most complete exercise intervention to incorporate for entire populations of people suffering obesogenic diseases. Incorporating both styles of exercise allows individuals to fully develop relevant energy systems, adapt their metabolic condition, while allowing for spontaneity during program design and a larger sense of ownership of their health.

Careful considerations are needed for exercise variables to ensure competency (e.g., exercise selection and execution, volume, frequency, etc.). A fine balance is drawn between using minimum effective dose of training across global muscle groups to enhance the benefits of RT while maximizing the impact of the exercise on the individual. For example, AT had shown to be an effective treatment for reducing cardiometabolic risk factors in applications where participants were not directly supervised [16]. This could prove beneficial when developing exercise prescriptions for communities as only focusing on directly supervising RT and not for AT would reduce significant hours of work for the researchers.

Importantly, several characteristics are required to keep large groups of participants engaged in accomplishing health target goals. For example, extensive counseling was shown to build healthy patterns during the intervention and increase the likelihood behavioral changes would be lasting [16]. Second, using direct lines of communication to bring about the awareness for the development of metabolic dysregulation while also simultaneously working on improving those conditions is vital. Finally, improving the level of knowledge of the community surrounding the health outcomes of interest would work exponentially to improve the total health rather than working individually with people in need.

## Future Directives

Research is needed to explore the efficacy and validity of large-scale exercise intervention programs to combat the obesity pandemic. Funding and staffing these studies will be challenging as the use of multiple fitness facilities and exercise professionals will be needed to service single communities. Because of these constraints, few large population-based studies have been conducted. Thus, unrealized issues may remain buried until we begin to organize, conduct, and analyze these interventions.

Ultimately, these exercise intervention studies will establish the validity and effectiveness of utilizing exercise as a treatment for the obesity epidemic. With the hope that medical institutions can begin to incorporate direct exercise interventions for people suffering from metabolic disease with the highest level of efficacy. Exercise is the best medicine available to prescribe globally to people in critical need of reversing the growing trends in poor health status seen in the majority of today’s societies. Combining exercise prescription alongside proper medical care provides a scope of care needed to effectively combat the disease cycle and allows for clinicians to meaningfully impact patient’s life in longitudinal manner. The ability to use exercise as a medical prescription extends not only to directly treating the symptoms of the diseases but also to address underlying behaviors and knowledge gaps that may be the cause of the symptoms.

Italy provides an excellent model of diabetic care that could foster the birth and growth of similar facilities more broadly aimed at reducing obesity and incidence of obesogenic diseases. The use of 680 outpatient diabetic care centers, all attached to a medically supervised fitness facility, spread throughout the country provides a glimpse at the scale necessary for combating the expansion of metabolic disease [18]. This example also points to a limitation that will be seen in bringing this concept to other areas of the world, as Italy and other countries that were included in this study institute public health care [6,9,11–15]. There could be unknown challenges in recruitment strategies, adherence, and foundation programs similar to the Italian outpatient diabetic clinics in a private health insurance environment such as the USA.

Exercise as a medicine is only as impactful as the manner in which the exercise is prescribed and executed, therefore lending the need to have medical facilities to ensure proper application of treatment. The use of a collaborative network of clinical exercise facilities for use to those that have been prescribed exercise as medicine is a potential solution to the growing obesity epidemic [19]. The application of CT has shown to be a cost-effective strategy for combating T2DM as compared to standard pharmacological intervention and lifestyle recommendations which could allow for partnership between medical institutions and healthcare providers [19]. This use of exercise to combat the disease would reduce the economic burden not only for the individual but also for insurance companies covering proportion of the care assessed traditionally. This incentive alongside of federal and state government helping to subsidize community programs to provide a much-needed service to public health.
